# Micropatterning of Substrates for the Culture of Cell Networks by Stencil-Assisted Additive Nanofabrication

**DOI:** 10.3390/mi12010094

**Published:** 2021-01-18

**Authors:** Anita Previdi, Claudio Piazzoni, Francesca Borghi, Carsten Schulte, Leandro Lorenzelli, Flavio Giacomozzi, Alessio Bucciarelli, Antonio Malgaroli, Jacopo Lamanna, Andrea Moro, Gabriella Racchetti, Alessandro Podestà, Cristina Lenardi, Paolo Milani

**Affiliations:** 1 CIMaINa and Dipartimento di Fisica, Università degli Studi di Milano, Via Celoria 16, 20133 Milano, Italy; anita.previdi@unimi.it (A.P.); claudio.piazzoni@unimi.it (C.P.); francesca.borghi@unimi.it (F.B.); carsten.schulte@unimi.it (C.S.); alessandro.podesta@unimi.it (A.P.); cristina.lenardi@unimi.it (C.L.); 2 Center for Materials and Microsystems (CMM), Bruno Kessler Foundation (FBK), Via Sommarive 18, 38123 Trento, Italy; lorenzel@fbk.eu (L.L.); giaco@fbk.eu (F.G.); bucciarelli@fbk.eu (A.B.); 3 Center for Behavioral Neuroscience and Communication (BNC), Università Vita-Salute San Raffaele, Via Olgettina 58, 20132 Milano, Italy; malgaroli.antonio@unisr.it (A.M.); lamanna.jacopo@hsr.it (J.L.); andrea.moro@unimi.it (A.M.); racchetti.gabriella@hsr.it (G.R.)

**Keywords:** micropatterns, nanofabrication, nanostructured zirconia, primary cell networks, cell confinement, astrocytes

## Abstract

The fabrication of in vitro neuronal cell networks where cells are chemically or electrically connected to form functional circuits with useful properties is of great interest. Standard cell culture substrates provide ensembles of cells that scarcely reproduce physiological structures since their spatial organization and connectivity cannot be controlled. Supersonic Cluster Beam Deposition (SCBD) has been used as an effective additive method for the large-scale fabrication of interfaces with extracellular matrix-mimicking surface nanotopography and reproducible morphological properties for cell culture. Due to the high collimation of SCBD, it is possible to exploit stencil masks for the fabrication of patterned films and reproduce features as small as tens of micrometers. Here, we present a protocol to fabricate micropatterned cell culture substrates based on the deposition of nanostructured cluster-assembled zirconia films by stencil-assisted SCBD. The effectiveness of this approach is demonstrated by the fabrication of micrometric patterns able to confine primary astrocytes. Calcium waves propagating in the astrocyte networks are shown.

## 1. Introduction

The in vitro fabrication of cell networks able to simulate the basic elements constituting brain circuits and to maintain their native connectivity is of strategic importance for the understanding of brain circuits’ physiology. An emerging field is bottom-up neuroscience, in which basic cellular elements of the brain are thoroughly analyzed to understand the functioning mechanism of higher-level circuits and eventually of the brain as a whole [1,2,3]. This approach is rewarding, but the interpretation of results is not straightforward, because a functional dissection of each single elementary module from its interacting counterparts is needed. One way around it is to assemble tailored cell networks in vitro by culturing cells on substrates specifically engineered to restrict neural cell adhesion to specific areas that match the topology of simple neural networks [4,5,6]. Such simplified systems of brain cells represent a tool to perform functional studies as it was demonstrated that small cultures on grid networks yield electrophysiological properties similar to random, brain-scale preparations, despite their unique topology and connectivity [5].

Substrates with controlled topography and chemical composition at the micro- and nano-scale are considered very effective platforms for the study of complex behavior such as neural cell signaling and to develop high-throughput protocols for drug screening and cell-based therapeutic solutions [7,8,9,10]. In addition, surfaces with well-defined nanotopographical properties that mimic the ones present in natural extracellular matrix (ECM) are of particular interest, in the field of mechanobiology, to explore mechanotransduction-dependent modulations of cell phenotype development, adhesion, differentiation, motility, and apoptosis [11,12].

Several methods for substrate micro- and nano-patterning are based on the selective modification of the surface chemistry to create cell-adhesive/repelling regions [11,12,13]. Among the most widespread methods for chemical patterning, micro-contact printing (MCP) [14] relies on the pattern transfer of the ink of interest from a soft stamp, usually made of polydimethylsiloxane (PDMS) or poly(methyl methacrylate) (PMMA) to a substrate. The master mold is usually a silicon wafer microfabricated with photo-resist lithography (PRL). This technique is simple and flexible towards the choice of substrate and of the ink to be patterned. On the other hand, the fabrication of the master mold is expensive, and the complex multi-step process is time consuming [8,12].

An alternative solution to MCP exploits commercial inkjet printers with simple hardware modifications to create fouling/anti-fouling patterns by directly jetting the chemicals of interest on suitable substrate. This is a low-cost solution for large-scale printing, however, only inks with certain fluidic properties can be printed and the smallest lateral resolution achievable is usually 100 μm. Resolutions down to 1 μm can be achieved with complex and expensive hardware modifications [8,12].

An approach transversal to different fabrication methods is stencil-assisted patterning (SAP). Soft or rigid stencil masks can be fabricated with different techniques (e.g., PRL, ion beam milling, laser cut, chemical etching). Different physical, chemical, or physico-chemical techniques are then used to deposit active species through the mask. Advantages and limits depend on the mask fabrication and deposition techniques used to obtain a certain lateral resolution [8,12].

Topographical patterns at the microscale can be obtained by using top-down subtractive technologies typical of silicon-based MEMS production. Fabrication of simple basic motifs such as grooves, pillars, dots with different dimensions, and pitches has been reported in order to reproduce and to recapitulate the elemental topographical cues that may influence the cell behavior [15,16]. In general, these high-precision fabrication methods have the advantage to be scalable, although with some difficulties. On the other hand, they are quite expensive and basically limited to silicon substrates. Most importantly, it is yet to be demonstrated that starting from simple topographical motifs one can realistically reconstruct the ECM topographical complexity that mediates all the interactions between the cell and its environment [16,17] that are of particular importance also in the neuronal context [18]. In fact, ECM topography is based on a very complex and random entanglement of nanoscale fibers and crosslinked reticular structures [19].

During the last decade, we developed an additive method to fabricate surfaces with multiscale controlled disorder quantitatively mimicking the nanoscale topography of biological systems [20,21,22,23]. We concentrated on titania and zirconia surfaces, because of their biocompatibility and their widespread use as implant and prosthetic materials [24,25]. Our bottom-up fabrication method is based on supersonic cluster beam deposition (SCBD) to produce nanostructured films with a nanoscale topography whose roughness can be accurately and reproducibly controlled and varied [23,26,27,28]. This allows us to fabricate substrates that mimic the intricated morphological characteristics of the ensemble of nanoscale components making up the ECM [23]. The precise control over nanoscale topography can be easily obtained over macroscopic areas, as it is required for the large number of experiments typical of in vitro biological assays, and compatible even with exigent (phospho)proteomics-based approaches [29]. SCBD allows to fabricate nanostructured films with a controlled morphology at the nanoscale. Standard physical vapor deposition techniques based on atom assembling for thin film deposition produce microcrystalline structure with no control on the nanoscale surface morphology, as discussed in detail in References [27,28].

Here, we report the high-throughput fabrication of micropatterned nanostructured substrates based on SAP, which can confine cells. The peculiarity of our approach is that it enables both cell confinement and replication of an ECM-like morphology on the substrate. This methodology essentially consists of two steps: an anti-fouling molecule is applied to a flat substrate, such as silicon or glass, in order to create a cell-repellent monolayer; then, a nanostructured zirconia (ns-ZrO_x_) coating is added via SCBD through stencil masks to create the cell-adhesive regions with controlled nanoscale roughness. 

We previously showed that this approach is valid in Reference [30], where we successfully confined a neuronal cell line, PC12, and primary hippocampal neurons on ns-ZrO_x_ dots of 150 μm diameter. In this work, we demonstrate that it is possible to fabricate much more complex patterns formed by interconnected microstructures, with features as small as tens of micrometers. Moreover, we obtained cell confinement with primary astrocytes, a type of glial cells able to grow elongated processes and known to be particularly adaptive [31,32]. We also report the observation and characterization of calcium waves propagating in a confined astrocyte network as a proof-of-principle of the effectiveness of our method. 

## 2. Materials and Methods

### 2.1. Substrate Fabrication

**Figure 1:**
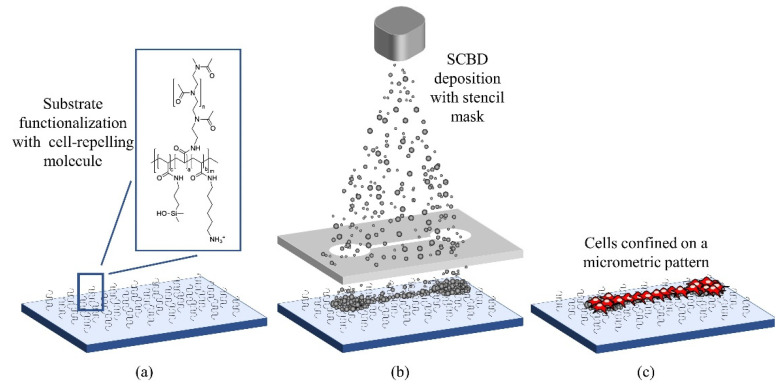
Schematic representation of the fabrication process of substrates for cell confinement.(**a**) The substrate is functionalized with the cell repelling copolymer PAcrAm-g-(PMOXA, NH2, Si). (**b**) A micropatterned film of ns-ZrO_x_ is deposited via Supersonic Cluster Beam Deposition (SCBD). (**c**) Cells are plated on the substrates and they adhere only to the ns-ZrO_x_ pattern.

The main steps for substrate fabrication are summarized in Figure 1: (a) an anti-fouling molecule is grafted to the surface of a glass substrate, (b) a nanostructured zirconia coating is deposited via SCBD through stencil masks, to create the cell-adhesive regions, and (c) the result is a selectively antifouling substrate where cells attach only on the adhesive areas. 

The antifouling molecule is the copolymer PAcrAm-g-(PMOXA, NH2, Si) that binds covalently to the hydroxylated surface [33] without affecting its roughness significantly, see Figure S1 in the Supplementary Information. In the latter, the detailed protocol for the substrate cleaning and functionalization is reported.

Nanostructured zirconia patterns are deposited by a SCBD apparatus equipped with a pulsed microplasma cluster source (PMCS). The details of nanostructured film fabrication process can be found in References [27,28,34]. 

**Figure 2:**
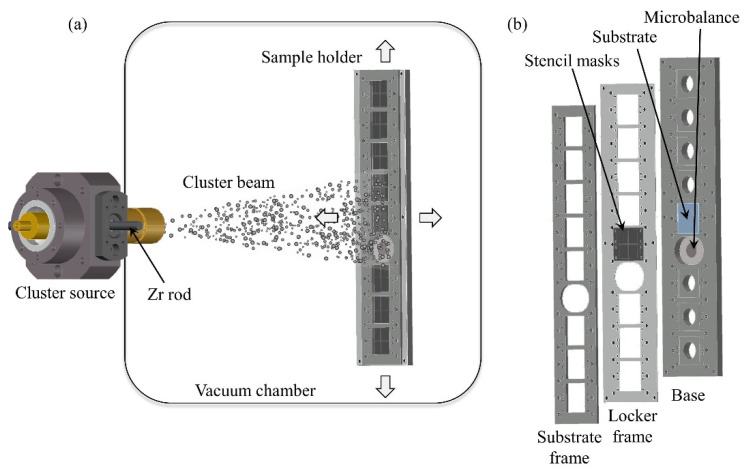
(**a**) Schematic representation of the SCBD apparatus (not to scale): the cluster beam is produced by the pulsed microplasma cluster source (PMCS) equipped with a zirconium rod and it impinges on the sample holder. By rastering the sample holder, a patterned film is deposited on all the samples. (**b**) Design of the sample and stencil mask holder. The three parts ensure the right positioning of the stencil masks with respect to the deposition substrates.

Figure 2a reports a schematic representation of the SCBD apparatus. In brief, in the PMCS, a zirconium rod is ablated via an aerodynamically confined plasma discharge ignited after the injection of a high-pressure Ar pulse [35]. The ablated species thermalize with the injected gas and condense to form zirconia clusters inside the source cavity. The latter is connected through a nozzle to a high-vacuum chamber [36]. The cluster/Ar mixture expands into vacuum to form a supersonic seeded beam that impinges on the substrates that are mounted on a sample holder placed at the center of the vacuum chamber (Figure 2a), thus forming a nanostructured zirconia film. Since the nanoscopic roughness of the film is directly proportional to its thickness, we can reproducibly control and tune the surface nano-topographical properties of the cluster-assembled films [27].

A sample holder hosts the deposition substrates (here glass substrates) and the stencil masks (Figure 2b): the sample holder comprises a base and locker frame, fabricated with mechanical machining of aluminum slabs, holding substrates and stencil masks together, and a substrate frame that ensures the substrates’ position. A 4-axis motorized manipulator allows the rastering of the sample holder in order to obtain a uniform deposition over an area of 200 × 40 mm. The holder hosts a quartz microbalance to monitor the deposition rate of zirconia clusters. 

### 2.2. Stencil Mask Fabrication and Characterization

**Figure 3:**
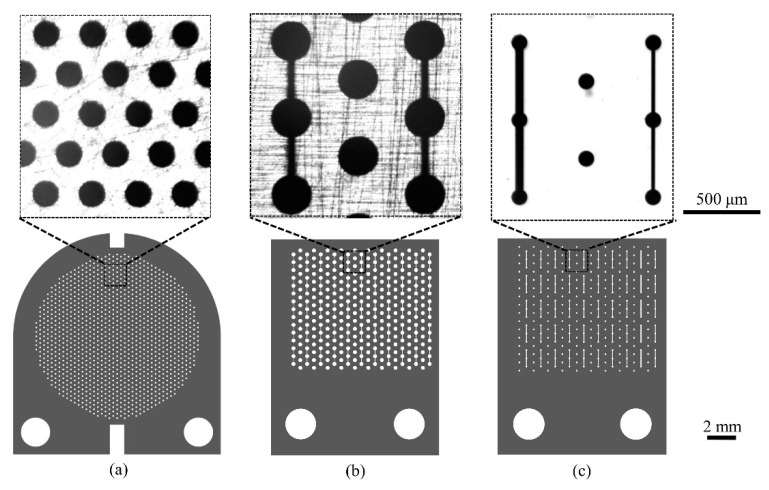
A selection of stencil masks used in this work: design of the mask (bottom) and phase contrast images of details of the pattern (top). (**a**) Stainless-steel mask patterned with dots of diameter 150 μm, interaxial distance 500 μm, 150 μm thin (mask A, Supplementary Table S1). (**b**) Stainless-steel mask patterned with dots and channels. The diameter of the dots is 250 μm, the interaxial distance is 500 μm, and the channels are 20 μm wide. The mask thickness is 50 μm (mask Q13, Supplementary Table S1). (**c**) Silicon mask patterned with dots and channels. The dots’ diameter is 100 μm and the interaxial distance is 500 μm. Alternating lines of dots are connected by 20 or 50 μm wide channels. The patterned area is 100 μm thin (mask Q19, see Supplementary Table S1).

We used stencil masks fabricated with two different techniques: laser cutting (LC) of thin stainless-steel foils and photo-resist lithography (PRL) of silicon wafers. Different pattern designs were evaluated (sub-millimetric dots with or without micrometric channels), and some examples are reported in Figure 3. LC steel masks with dots were purchased from Lasertech Srl (Cernusco sul Naviglio, Milan, Italy), and LC steel masks with dots and micrometric channels were obtained from Kirana Srl (Rovereto, Trento, Italy). The detailed characteristics of the masks with the main features of the stencil patterns are listed in Table S1 of the Supplementary Information. 

LC stainless-steel masks present several advantages: they are cheap, easy-to-handle, and, in principle, reusable indefinitely, provided that an effective cleaning protocol can be established (see the Supplementary Information). 

**Figure 4:**
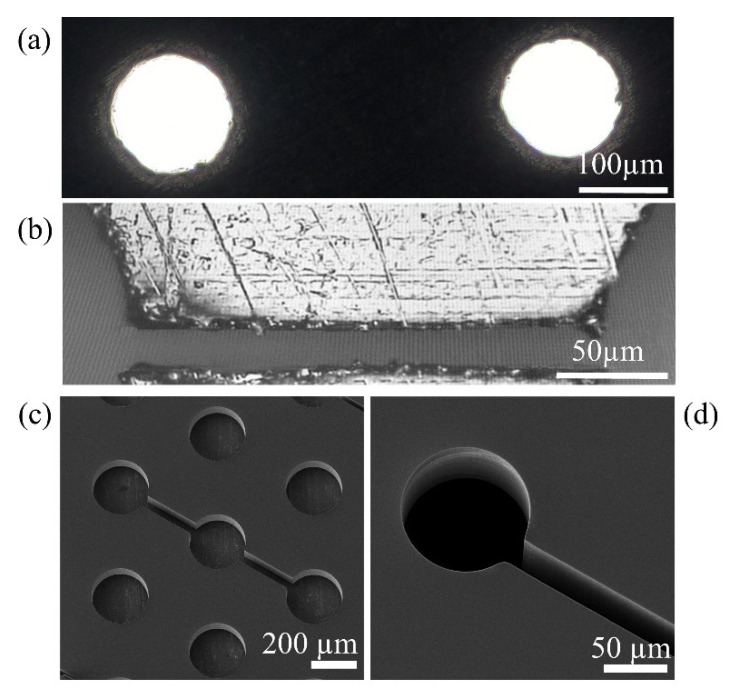
Details of stencil masks produced with LC of stainless-steel foils and with photo resist lithography (PRL) of silicon. (**a**) Optical micrograph of 150 μm wide holes of LC steel mask with jagged borders (mask A, Supplementary Table S1). (**b**) Optical micrograph of a 20 μm wide channel of LC steel mask with a re-solidified drop of metal closing-up the channel opening (mask Q13, Supplementary Table S1). (**c**) SEM image of 250 µm wide dots with an interaxial distance of 500 µm. The channels connecting the dots are 50 µm wide (mask Q19, Supplementary Table S1). (**d**) SEM image of 100 µm wide dot with a 20 µm wide channel (mask Q16, Supplementary Table S1). Images (**c**,**d**) were acquired using a SEM (VEGA3 TESCAN) with a 10 kV electron beam at different magnifications.

Concerning precision and pattern reproducibility, the LC technique suffers from fabrication defects due to, for example, the re-solidification of drops of metal or loss of planarity of the masks, due to inefficient heat dissipation. Figure 4a,b report optical micrographs of LC stainless-steel masks (the thickness is respectively 150 and 50 μm) with defects like jagged opening borders. 

Meanwhile, PRL of silicon allows the manufacture of stencil masks with a high level of precision: features with dimension in the range of a few μm are accurately reproduced. These stencil masks were fabricated starting from a 6″ (100) silicon wafer. By using Deep Reactive Ion Etching (DRIE), the features defined by lithography were etched on the silicon frontside, and then, by removing a wider area from the backside, a membrane thickness of 100 μm was obtained. Figure 4c,d show a selection of scanning electron microscope (SEM) images of details of PRL silicon masks. There are no visible fabrication defects in any pattern detail and the openings’ borders are smooth. The 20 μm large channel of Figure 4d is produced with no defects. PRL silicon masks are more expensive and much more fragile than LC stainless-steel masks.

### 2.3. Morphological and Optical Characterization 

Atomic force microscopy (AFM) was used to characterize the surface morphology of the substrates and of the films, using a Multimode 8 microscope produced by Bruker. We acquired 5 images of extension 2 × 1 µm of the samples in order to characterize the morphologies of the nanostructured zirconia at the nanoscale. AFM was operated in air in tapping mode, using silicon nitride cantilevers mounted onto single-crystal silicon tips with a nominal radius < 10 nm, a resonance frequency in the range of 250–400 kHz, a scan rate of 1 Hz, and a sampling resolution of 2048 × 512 points. The images were flattened by line-by-line subtraction of first- and second-order polynomials in order to remove artifacts, due to the sample tilt and the scanner bow. From flattened AFM images, the root-mean-square surface roughness (R_q_) was calculated as the standard deviation of the surface heights.

The phase contrast images were taken with a microscope (Axiovert 40 CFL, Zeiss, Oberkochen, Germany) equipped with 20×/0.3 ph1, CP-ACHROMAT 10×/0.25 Ph1, 5 × /0.12 CP-ACHROMAT objective and with a high definition photo camera (True Chrome HD II, TiEsselab) operated by ISCapture imaging software.

### 2.4. Cell Culturing

In this work, we used primary astrocytes derived from the hippocampus of neonatal Sprague-Dawley rats (Charles River Laboratories Italia). All the procedures were performed according to the research and animal care procedures approved by the institutional animal care and use committee for good animal experimentation of the Scientific Institute San Raffaele complying with the code of practice for the care and use of animals for scientific purposes of the Italian Ministero della Salute (Ministry of Health) (IACUC No. 728).

After extraction, the cells were maintained in MEM medium supplemented with 10% fetal calf serum, 33 mM of glucose, 2 mM of Glutamax, and 2 U/mL of Penicillin-Streptomycin (all reagents were obtained from Thermo Fisher Scientific, Gibco Massachusetts, USA, if not stated otherwise). Cells were grown on standard culture substrates for 12 days in vitro (DIV) at 37 °C and 5% CO_2_, and every 3 days, the culture medium was replaced. Cells were then detached with a trypsin/EDTA solution and, after centrifugation (1000 rpm, 4 °C for 5 min), the pellet was resuspended. The cells were subsequently seeded on the patterned substrates with a density of 12,500 cells/cm^2^. On these substrates, cells were kept in the same culture medium described above for 1 day, replacing the medium the day after. At day 2, the medium was replaced with one with a 1% concentration of fetal calf serum. The following days, the medium was replaced every 3 days. The immunofluorescence images were taken after 3 DIV, whereas the calcium imaging experiments were performed after 6 DIV.

### 2.5. Immunofluorescence Imaging

All the reagents were purchased from Merck KGaA, Darmstadt, Germany, if not stated otherwise.

For the immunofluorescence imaging, we used astrocytes grown on our patterned substrates (dots and micrometric bridges, produced with silicon stencil masks) for 3 days. We fixed them with 4% paraformaldehyde (PFA)/phosphate buffered salin (PBS) for 10 min. We then permeabilized the cell membranes with 0.2% Triton X-100/PBS for 3 min and blocked with 3% bovin serum albumin (BSA)/PBS. Phalloidin, tetramethylrhodamine (TRITC) conjugated was used to stain the actin cytoskeleton of the cells and Hoechst 33,342 for the nucleus. They were incubated for 45 min in a humid environment at room temperature. After the staining, the cells were mounted with ProLong^®^ Gold antifade (MolecularProbes).

Images were taken with a confocal microscope (Nikon A1R) with objectives 4×, 10×, and 20× at the UNI^TECH^ NOLIMITS Imaging facility of the University of Milano.

### 2.6. Calcium Imaging

Hippocampal astrocytes grown for 6 days onto a patterned film (100 μm dots connected by 50 and 20 μm wide bridges, produced with a silicon mask) were loaded with 2 μM Fluo-4 AM (Thermo Fisher Scientific, Invitrogen, Waltham, MA, USA) for 30 min at 37 °C, 5% CO_2_. During the experiment, the cells were kept in air, at room temperature, and submerged in Tyrode. This solution contains 119 mM NaCl, 5 mM KCl, 2 mM CaCl_2_, 2 mM MgCl_2_, 25 mM 4-(2-hydroxyethyl)-1-piperazineethanesulfonic acid (HEPES), and 30 mM d-glucose, the pH was adjusted to 7.4 with NaOH, and the osmolarity was adjusted to 300 mOsm. O_2_ was bubbled into the solution for the duration of the experiment. The sample was mounted on a customized holder with tubes enabling constant replacement of the Tyrode solution.

The cells were stimulated with a 100 mM l-Glutamic acid solution (monosodium salt, acquired from Sigma Aldrich, Saint Louis, MO, USA). Droplets of the stimulating solution of volume < 4 pL were delivered with a pressure application device (PDES-01T, npi electronic, Tamm, Germany) equipped with glass micropipettes pulled to have a tip diameter of approximately 3 μm. To wash away the stimulation solution right after its application and limit direct stimulation to few cells located in a restricted area, we used three parallel pipettes of diameter 500 μm filled with Tyrode that ensured a constant flow of solution washing away the glutamate at the stimulated site.

We recorded several 5-min time-lapse fluorescence videos with a microscope (Axiovert 135, Zeiss, Oberkochen, Germany), equipped with a standard filter set for fluorescein isothiocyanate (FITC) and a digital camera (Orca-ER, Hamamatsu). 

We analyzed the videos with custom-made scripts developed in MATLAB (2020b, The MathWorks Inc., Natick, MA, USA). The fluorescence traces were extracted as a sum of the intensity of pixels within circular ROIs (Regions of Interest) of equal radius, with the center corresponding to the most responsive cell somas, located manually. Each trace was normalized by the baseline intensity value, and a linear background was subtracted. We also performed a 10-point box-smoothing of the traces to reduce the high-frequency noise.

## 3. Results and Discussion

### 3.1. Replication of Micrometric Stencil Masks’ Features

SCBD is particularly effective for the fabrication of micropatterned thin films exploiting stencil masks [37]. The high degree of collimation, typical of supersonic expansions, allows large lateral resolution in the replication of features down to tens of micrometers. The use of aerodynamic lenses allows control on the cluster transverse velocity and mass distribution, with no significant loss in beam flux [38]. The result is a highly focused beam, with a strong intensity gradient decreasing from the center to the periphery, which results in the deposition of films with a relevant radial thickness variation over small areas, typically on the millimetric scale [39].

Films with a homogeneous thickness over large areas can be obtained by rastering the deposition substrates in the plane perpendicular to the beam axis. This allows averaging out the radial intensity dependence. However, rastering reduces the accuracy of reproduction of the stencil mask pattern. In fact, every portion of the deposition substrate will receive clusters impinging with trajectories whose angles with respect to the normal span from zero to the divergence angle of the beam. The penumbra effect around the edges of the patterned deposits causes blurred borders due to clusters overcoming the area located underneath the stencil mask opening. 

**Figure 5:**
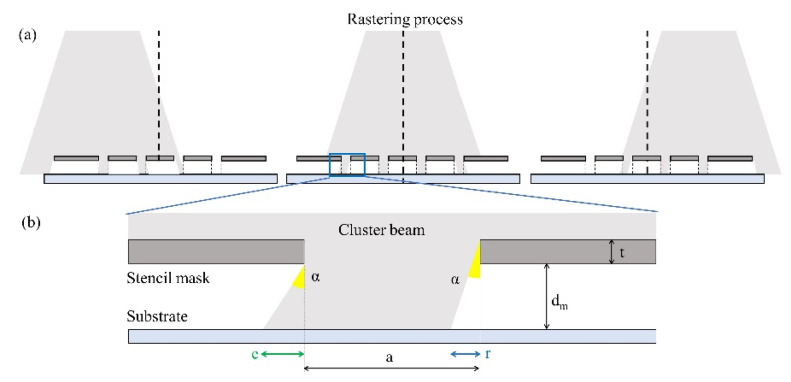
Schematic representation of a beam passing through a stencil mask and impinging on a substrate. (**a**) Rastering process: three different source–substrate relative positions are displayed, in which each opening intercepts a portion of the beam with a different inclination. (**b**) Single mask opening of extension *a*. A portion of the cluster beam passes through a stencil mask of thickness *t* with angle α and impinges on a substrate at a distance *d_m_* from the mask. Due to the non-zero inclination of the beam with respect to the normal to the substrate plane and the presence of a gap between the mask and the substrate, the beam overcomes the pattern borders, producing an enlargement *e*. Also, the right side of the pattern is not completely covered, causing a reduction of extension *r* in the deposition.

Figure 5 reports a schematic representation of the deposition process. In the different source–substrate relative positions (Figure 5a), the inclination of the portion of the beam intercepted by each stencil mask opening varies. Figure 5b focuses on one opening of the stencil mask and highlights how the cluster beam overcomes the area under it, due to the non-zero distance between the mask and the substrate and to the inclination of the cluster beam. 

**Figure 6:**
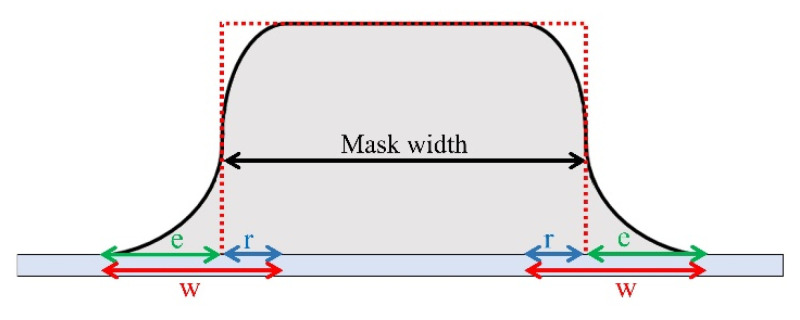
Schematic representation of patterned film height profile obtained with SCBD through a stencil mask, with a non-divergent beam (red dotted line) and with a divergent beam (black continuous line). The cluster beam divergence is responsible for a blurring effect. The lateral sides of the profile rise gradually, with a width *w*, given by the sum of the enlargement of the mask opening *e* and the width of the profile that does not reach the maximum height of the film, due to the penumbra effect, *r*.

Figure 6 reports the height profile of a patterned film resulting from a perfectly focused cluster beam, where the transverse velocity component of each cluster is negligible (red dotted line), and one resulting from a beam with clusters of different transverse velocities (black, continuous line). The blurring effect consists of both an enlargement of the pattern feature overcoming the borders of the stencil mask holes, in Figure 6 indicated with *e*, but also of a reduction in the thickness of the layer deposited close to the borders of the stencil mask holes, indicated by *r*. The sides of the latter have a shape that reflects the distribution of the transverse velocities of the clusters. The rising width *w* of the profile is given by the sum of the enlargement *e* and the reduction *r*.

The blurring effect can be explained with a purely geometric argument. In fact, with a beam of maximum divergence angle *α_max_* and a stencil mask of thickness *t* at a distance *d_m_* from the deposition substrate, the amplitude of this rising width *w* can be calculated as:

*w* = *e* + *r* = (2 *d*_*m*_ + *t*) *tan*(*α*_max_) (1)

In order to increase the step sharpness, one should produce a more focused cluster beam, for example, by increasing the supersonicity of the beam [40]. One should also reduce as much as possible the mask-substrate *d_m_* distance and use thin stencil masks (i.e., reduce *t*). In this specific case, the measured divergence angle of the cluster beam is α_max_ = (5 ± 1)°, the silicon mask thickness corresponds to *t* = (100 ± 1) μm, and the estimated mask–substrate distance is *d_m_* = (50 ± 10) μm. With these values, according to Equation (1), we expect a rising width *w* = (17.5 ± 5.9) μm.

**Figure 7:**
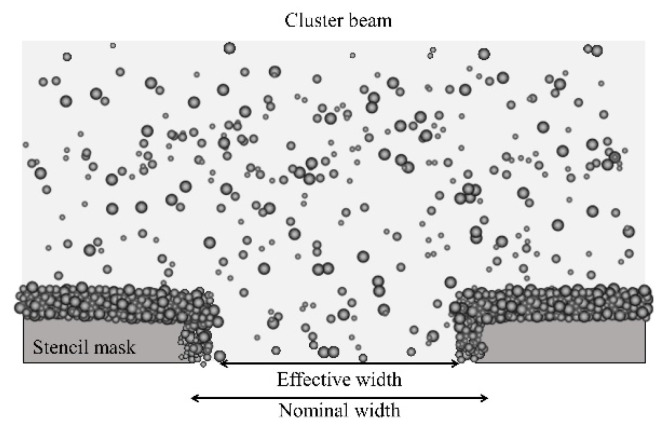
Schematic representation of the clogging effect due to accumulation of zirconia clusters inside the stencil mask’s opening: the nominal width of the stencil mask opening is progressively reduced to a smaller effective width as the deposition is carried out.

The finite distribution of transverse velocity of the clusters in the supersonic beam is an additional source of pattern distortion. In fact, clusters impinging on the lateral borders of the stencil masks’ openings tend to accumulate progressively during the deposition, causing a clogging effect of the stencil mask pattern. Figure 7 schematically shows how the stencil mask opening’s diameter gradually decreases as more and more material is deposited on the borders.

This produces a progressively conical shape of the deposit. The rising width *w* is further increased, as the contribution of the clogging effect is summed with the geometric blurring, as described above (Equation (1)). The clogging effect’s contribution to *w* is smaller than the thickness of the deposited film, but it is relevant when the film thickness is comparable to the lateral dimensions of the stencil mask pattern features. In the present work, we deposited films with thickness below 210 nm, and therefore clogging is negligible.

Progressive mask clogging may represent a limit when fabricating patterned films of highly homogenous thickness: the extension of the homogeneous area imposes a limit of the film thickness. On the other hand, as Barborini et al. showed in Reference [40], this effect can be exploited to fabricate nanostructured three-dimensional (3D) objects with controlled geometrical properties, such as nanostructured tips. 

Lastly, the quality of the stencil masks is crucial for the accurate reproduction of stencil patterns on substrates. Any defect in fabrication of the stencil mask will be transferred on the patterned film in an amplified manner due to the clogging effect. Among the different approaches available for the fabrication of stencil masks, we tested two different types of masks: LC stainless-steel foils and silicon sheets patterned with PRL. Stencil masks should be as thin as possible and non-deformable to guarantee a constant separation between the mask and the substrate over the whole deposition area. 

Due to their fabrication defects, steel stencil masks are suitable for patterns with features of dimensions of the order of 100 μm and when the requirements of pattern transfer concern the extension of the pattern feature areas and not the fine micrometric details of the pattern. The jagged borders of the stencil masks do not compromise the overall pattern topology as they are much smaller than the mean dimension of the pattern features (see Figure S2 in the Supplementary Information). 

When the width of LC steel mask’s opening is of a few tens of micrometers, the effect of the edge defects becomes more relevant. This is particularly evident, for example, in the pattern given by dots connected by 20 μm wide bridges. The width of the border of the micro-bridges has wiggles of dimensions comparable to the bridge width, therefore the topography is severely affected (see Figure S3 in the Supplementary Information). Defects like consistent wiggles and interruptions in channels were found to affect between 40% and 70% of the channels in each mask.

**Figure 8:**
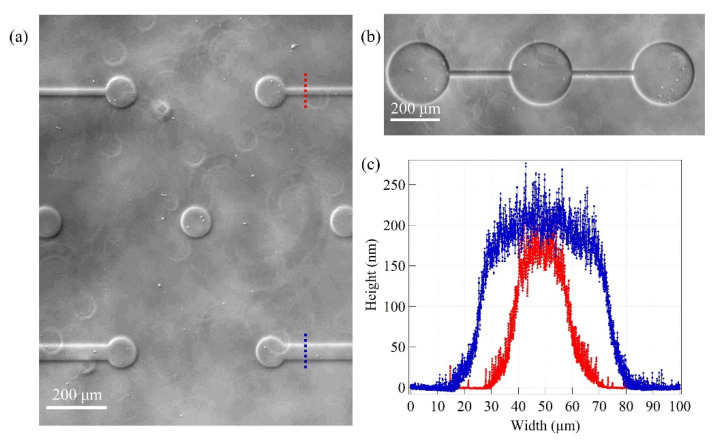
(**a**,**b**) Phase contrast images of ns-ZrO_x_ patterns obtained with PRL silicon stencil masks. In (**a**) the diameter of the dots is 100 μm, in (**b**) 250 μm (referring to Supplementary Table S1, the labels of the masks are respectively Q16 and Q19). Alternating rows of dots are connected by 20 and 50 μm wide bridges. (**c**) Atomic force microscopy (AFM) height profile maps of ns-ZrO_x_ micrometric bridges. The blue and red traces correspond to 50 and 20 μm wide bridges, respectively. The profiles were measured on different portions of the same sample: the thickness difference clearly visible in the two profiles can be attributed to the more effective blurring on the smaller mask openings.

Higher levels of accuracy in the reproduction of micrometric patterns can be obtained with PRL silicon masks. They present perfectly regular openings, in the micrometric range, so the transferred patterns are only affected by the defects deriving from the beam divergence. The phase contrast images reported in Figure 8a,b show zirconia patterns deposited with PRL silicon masks: the dots are circular, and their borders are smooth, with no wiggles. The bridges present no interruptions, and their width is constant over their whole extension. In Figure 8c, AFM height profiles of micrometric bridges of width 50 μm (blue trace) and 20 μm (red trace) are reported. Both profiles display a plateau area in the central zone of the bridge and a rising width, *w*, of ~20 μm, compatible with the expected value and irrespective of the channel width. This result is coherent with Equation (1), since the blurring effect does not depend on the width of the mask opening. Although the two height maps were measured on the same sample, the two microbridges have different plateau heights due to the blurring effect at the borders. Here, the width of the channel on the mask is comparable to the rising width of the nanostructured zirconia bridge; therefore, the whole pattern width is affected by blurring. The maximum height of the deposit cannot reach the value expected in an un-patterned area. 

### 3.2. Nanostructured Morphology of the Micropatterns 

The possibility of controlling the zirconia morphology at the nanoscale [36] and its homogeneity on the whole patterned sample is of pivotal importance since a small change in the morphological properties of the substrate can determine different functional properties of the thin film and promote specific mechanotransductive signals to the cells [20,21,29,41].

**Figure 9:**
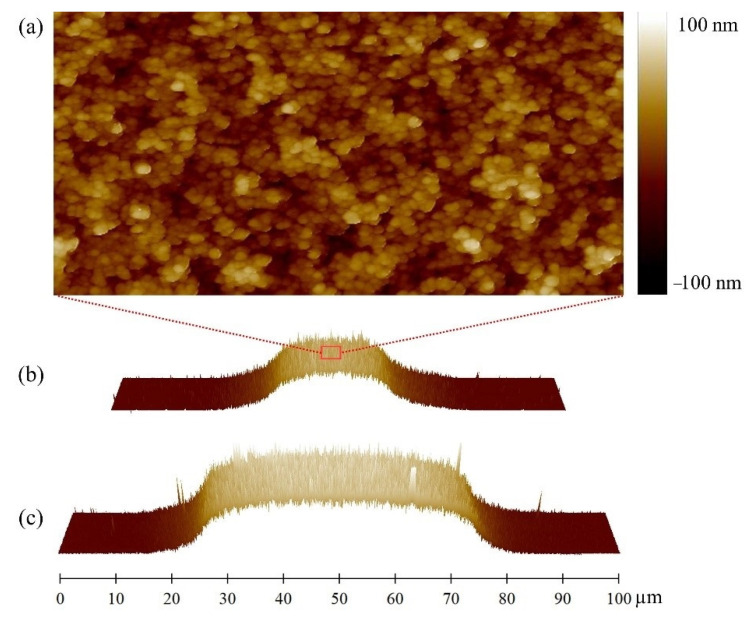
(**a**) Representative AFM topographical map (2 × 1 μm) of nanostructured zirconia in the center of a 20 μm large bridge, z-scale ranges from −100 to 100 nm. (**b**,**c**) Three-dimensional (3D) AFM maps of 50 and 20 μm large bridges, respectively.

Figure 9 shows the AFM images of the nanostructured zirconia of micrometric bridges characterized by different widths (20 and 50 μm). As mentioned before, when the width of the mask channel is comparable to the dimension of the lateral height gradient of the nanostructured zirconia bridge (as is the case for the 20 μm wide bridge), the highest thickness of the deposited film, characterizing the central plateau, is lowered due to the blurring effect.

The resulting surface roughness, *R_q_*, also decreases, since it depends on the film thickness, *t*, according to a simple scaling law, *R_q_* ~ *t^β^* [42], where the growth exponent, *β*, is 0.4 [36].

The morphological properties of nanostructured zirconia in different regions of the micrometric patterns are summarized in Table 1. 

In biological applications, it was demonstrated that substrates with different nano-roughness provide distinct biophysical stimuli that can have a direct impact on mechanotransducive cellular structures (such as the integrin adhesion complexes and the cytoskeleton) and eventually cell development and functioning [18,20,21,29].

### 3.3. Cell Confinement 

The effectiveness of our approach in cell confinement with patterned substrates has already been demonstrated with a neuronal cell line, PC12, and with primary hippocampal neurons [30].

Here, we use primary hippocampal astrocytes, a type of brain cells whose biological function has recently been demonstrated to be closely related to neuron signaling [31]. Previously believed to have a mere supportive role in the central nervous system, there is now evidence that astrocytes participate actively in synaptic transmission. The concept of tripartite synapse has been developed to describe the spatial proximity but also functional relation between pre- and post-synaptic neurons and astrocytes at the synaptic level [43]. 

On standard culture substrates, astrocytes grow in unorganized, interwoven monolayers, in which it is challenging to discriminate the functional connections between adjacent cells. Also, a uniform layer of cells hardly compares to the complex anatomical structures in which astrocytes are found in vivo.

Confining these primary astrocytes is particularly challenging as they can grow elongated processes and express a wide variety of cell adhesion molecules [32,44]. Astrocytes accordingly display a remarkable adaptive plasticity and adhere to many different substrates, allowing, e.g., the interaction with the brain interstitial matrix and the basement membrane in the vicinity of brain capillaries, or to enwrap neuronal components’ surfaces [31,44]. Substrates must be fabricated exploiting a combination of materials with robust and stable in time cell-adhesive/repelling properties. Here, we show that the patterned cluster-assembled zirconia substrates represent an effective platform to constrain adhesion of astrocytes onto specific areas and make them follow specific designs determined a priori. We underline that the zirconia films are used in their original state, with no further functionalization to enhance adhesion (such as poly-L-ornithine and matrigel) prior to cell plating. 

**Figure 10:**
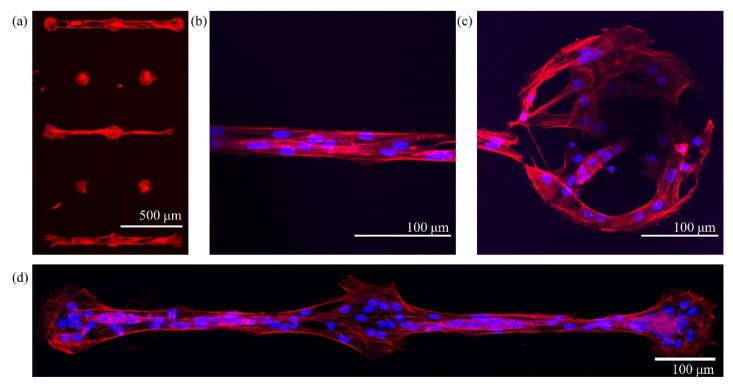
Immunofluorescence images of primary hippocampal astrocytes confined on patterned zirconia substrates, fixed and stained after 3 DIV. The actin cytoskeleton was stained with phalloidin, tetramethylrhodamine (TRITC)- conjugated, red) and the cell nucleus with Hoechst 33,342 (blue). (**a**) Actin cytoskeleton of astrocytes plated on alternating rows of isolated dots and dots connected by microbridges. In the first and fifth row, the microbridge is 50 μm wide, and in the third row, 20 μm. The dots have a diameter of 100 μm. (**b**) Focus on a 20 μm wide microbridge. (**c**) Focus on a dot of diameter 250 μm. The shapes of the cells adapt to the geometry of the portion of the pattern that they occupy, cells on bridges have an elongated shape and processes, oriented parallel to the direction connecting the dots. (**d**) A triplet of 100 μm diameter dots connected by a 20 μm bridge. This image was obtained by combining several 10× images.

We tested the patterns with micrometric resolution comprising zirconia bridges connecting dots, fabricated with the procedure described above and using PRL silicon masks. The immunofluorescence images of Figure 10 display the staining of the actin cytoskeleton (red, phalloidin TRITC conjugated) and of the nuclei (blue, Hoechst 33,342) of astrocytes at 3 DIV confined on patterns of dots connected by bridges of different dimensions.

In Figure 10a, a low-magnification image shows the actin cytoskeleton of cells in different confinement configurations, i.e., on triplets of dots connected by 20 and 50 μm wide bridges, and on isolated dots. Astrocytes grow only on the adhesive patterned areas and remain confined both on the single dots and on the interconnected triplets of dots. The images in Figure 10b, c show details of pattern features, a 20 μm bridge and a dot of 250 μm diameter, respectively. The image in Figure 10d was obtained by stitching together several 10× images (using the grid stitching plugin of ImageJ, NIH, New York, NY, USA). 

Interestingly, cells tend to take on the shape of the underlying pattern: cells on the borders of the dots adapt to their circular shape, whereas cells on the micrometric bridges are elongated toward the direction connecting the dots. On the micrometric bridge, an alignment of the cytoskeleton actin components in the direction parallel to the bridge is clearly visible.

Astrocytes are homogeneously distributed on the adhesive area of the samples, and very few cells adhere on the cell repelling area. A statistical analysis of the images, carried out by counting the nuclei of the cells (average over 5 images), showed that 93% of the cells adhering on the sample surface can be found on the ns-ZrO_x_ adhesive area portions, occupying only 6% of the total surface available. In more detail, 85 % of the cells are strictly inside the nominal dimension of the pattern feature, 7% are on the border, in contact with the cells within the patterned area, and only 7% of the cells actually adhered on the cell-repelling surface, with no contact with the cells on the ns-ZrO_x_ areas.

We are thus able to guarantee an optimum level of cell confinement and to determine a priori the geometry of the astrocyte network; potentially, we can also control the shape of the single cells. This is a particularly relevant result from the biological point of view: cells of the same type acquiring different shapes can show relevant physiological differences [45,46]. There is evidence suggesting that astrocytes do not have a homogeneous morphology throughout the brain, with potential implications regarding brain ageing and neurodegenerative diseases [47,48]; however, it is still not understood how astrocyte shape could be physiologically relevant. The possibility of controlling cell shape by fabricating ad hoc patterned substrates can provide a platform for a rich variety of experiments concerning cell morphology with astrocyte ensembles mimicking anatomical structures.

### 3.4. Testing the Functionality of the Network

Astrocytes can propagate signals in the form of calcium waves, mediated by channels called gap junctions. Moreover, synchronized calcium activity in astrocyte ensembles has been observed: this provides a mechanism for signal transmission in an organized functional network [49,50,51].

Cell patterning represents a viable strategy to assemble topologically controlled networks of astrocytes in vitro with a physiologically relevant geometrical shape. These specific platforms represent an important tool that enables the systematic study of calcium wave propagation in astrocytic networks, thus allowing a better understanding of the role of these cells in synaptic signaling in the central nervous system. 

We tested the functionality of the astrocyte network on our patterned substrates with calcium imaging, exploiting the Fluo-4 fluorescent indicator. This molecular probe signals the variation of intracellular calcium concentrations. We observed the propagation of calcium waves through neighboring cells both occurring spontaneously and elicited by the application of 100 mM glutamate solution droplets (volume < 4 pL). Astrocytes are known to respond to glutamate, inducing the opening of ion channels and the subsequent rise in cytoplasmic free calcium, which can propagate to neighboring cells [47].

**Figure 11:**
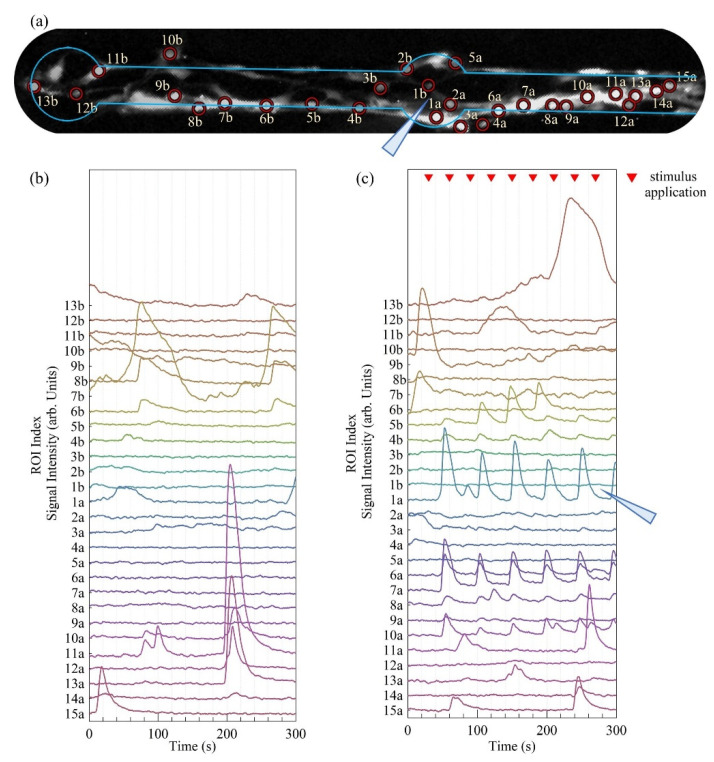
Calcium imaging of astrocytes confined on a micropatterned ns-ZrO_x_ sample. (**a**) Section of a fluorescent image of the calcium signal from astrocytes seeded on a sample patterned with dots of diameter 100 μm, 500 μm apart and connected by 50 μm wide bridges. Only two dots are included in the field of view. The border of the zirconia pattern is highlighted with a blue line. Red circles include each Region of Interest (ROI), corresponding to a cell. The labels report an index to allow the reader to identify the position of the cell with its calcium trace reported in the graphs below. The light blue triangle represents the micropipette delivering the stimulating solution. (**b**,**c**) Graphs reporting the calcium traces measured from the ROIs reported in image (**a**) when cells are non-stimulated and periodically stimulated with glutamate, respectively. The triangular markers on graph (**c**) indicate the application of the glutamate solution, every 30 s, and the light blue triangle represents the position of the pipette with respect to the cells. The indexes’ number increases for increasing distance of the cells from the pipette tip, and the letters a and b indicate whether cells are of the right or left side of the pipette.

In Figure 11a, we report a fluorescence image of the calcium signal from astrocytes seeded on a patterned substrate. Here, two dots of 100 μm diameter connected by a 50 μm wide bridge are depicted, the third dot is out of the field of view. The border of the zirconia pattern is highlighted with a blue line, and each ROI, corresponding to a cell, is signaled with a red circle and labelled with an index. The indexes are assigned so that ROIs labelled with a lower number correspond to cells closer to the micropipette, whereas “a” and “b” refer to cells on the right and left with respect to the micropipette. The light blue triangle represents the micropipette delivering the glutamate-stimulating solution. In Figure 11b,c, the fluorescent intensity time traces measured from each ROI are reported, with no stimulus (spontaneous activity) and with stimulus (elicited activity), respectively.

The stimulus consisted in the delivery of a droplet of glutamate solution every 30 s (the pressure application device was triggered every 30 s for 50 ms, thus delivering droplets of equal volume < 4 pL), and in the graph, this is indicated by the red triangular markers. In both graphs, the traces are ordered following the position of each ROI with respect to the direction parallel to the bridges, from left to right. The triangle representing the micropipette is reported in Figure 11c, between the traces of ROIs 1a and 1b, corresponding to the two cells closer to the stimulus. 

The fluorescence intensity traces registered without and with the periodic glutamate stimulus reveal a distinct behavior in the calcium activity of the astrocytic network. When the cells are not stimulated, spontaneous bursts of increasing intracellular calcium concentration take place and propagate to a few adjacent cells, see for example traces 10a, 11a, 12a, and 13a, which all present a peak at approximately 204 s. The burst is localized within 135 μm, the distance projected onto the direction parallel to the bridge between cell 13a and 10a. 

Instead, when the glutamate stimulus was applied, we observed a periodical rise in intracellular calcium in non-adjacent cells, and the stimulus propagated through cells on different sites of the bridge, further away from the micropipette opening. Traces relative to cells on both sides of the pipette, more than 370 μm apart (see, for example, 5b and 10a), all displayed peaks at similar times, proving that geometrically confined astrocytes behave as a functional network. 

This is a proof-of-principle that this approach provides a reliable method for the study of calcium waves in geometrically defined networks of astrocytes. A systematic characterization of the calcium propagation of cells grown of substrates with different nano-roughness or geometrical features of the pattern potentially mimicking the shapes of significant anatomic structures will be provided in future works. 

## 4. Conclusions

We developed a versatile and reliable experimental protocol to produce culture substrates for cell confinement onto different micrometric patterns, with an ECM-mimicking surface nanotopography. The nanostructured micropatterned zirconia films are deposited with the SCBD through stencil masks on glass substrates prepared with an anti-fouling treatment. Compared to other micro-fabrication techniques, this method provides cell confinement by exploiting a biocompatible material, with a nanoscale topography reproducing the natural environment of cells. Compatibly with the need of a large number of samples typical of biological experiments, our method is suitable for production on a large scale. 

In this work, we showed a strategy that enables to reproduce patterns with controlled topographical features both at the nanoscale (surface roughness) and at the microscale (micrometric pattern). Stainless-steel masks fabricated with the LC technology provided accurate results for patterns with features larger than 50 μm, whereas PRL silicon masks are suitable for the reproduction of patterns with features down to a few micrometers. The patterned zirconia films have a well-defined nano-morphology that can be accurately reproduced and varied to meet the requirements of the particular biological system under research. 

The effectiveness of our cell culture substrates was tested with primary hippocampal astrocytes: we found a stable confinement and adhesion of the cells on the predefined zirconia patterned areas with no further cell adhesion-enhancing functionalization. The versatility of the pattern design enables to systematically approach the investigation of the behavior of modular systems of cells, thus providing a platform for the in vitro reconstruction of complex architectures. 

Astrocytes grown on our patterned substrates display a rich calcium activity both spontaneous and elicited by glutamate application, as indicated by the propagation of a stimulated calcium wave along a micrometric bridge. This represents an important starting point for a systematic characterization of calcium signals in geometrically predefined astrocytic networks. 

## Figures and Tables

**Table 1:** Morphological properties of the nanostructured zirconia in different pattern features on the sample.

Geometry of the Pattern	Thickness (nm)	Roughness (nm)
Dot (250 μm diameter)	205 ± 5	19.5 ± 0.7
Bridge a (50 μm wide)	192 ± 5	19.0 ± 0.8
Bridge b (20 μm wide)	162 ± 4	16.5 ± 0.1

## Data Availability

Data is contained within the article or Supplementary Material.

## Supplementary Materials

The following are available online at https://www.mdpi.com/2072-666X/12/1/94/s1, Figure S1. Morphological AFM maps (2 × 1 µm) of glass coverslips, Table S1. Summary of the different types of masks tested in this work, Figure S2. Nanostructured zirconia dots, phase contrast image, and morphological AFM map, Figure S3. nanostructured zirconia pattern reproduced with a LC steel stencil mask, Figure S4. The clogging effect.
