# Supplementary material for: Micropatterning of Substrates for the Culture of Cell Networks by Stencil-Assisted Additive Nanofabrication
Source: Micromachines (Basel). 2021 Jan 18;12(1):94. doi: 10.3390/mi12010094 (PMC7829752; doi:10.3390/mi12010094)
Supplement: Supplementary file 1 [file micromachines-12-00094-s001.pdf]

micromachines

Supplementary Information: Micropatterning of Substrates for the Culture of Cell Networks by Stencil-Assisted Additive Nanofabrication

Anita Previdi [1], Claudio Piazzoni [1], Francesca Borghi [1], Carsten Schulte [1], Leandro Lorenzelli [2], Flavio Giacomozzi [2], Alessio Bucciarelli [2], Antonio Malgaroli [3], Jacopo Lamanna [3], Andrea Moro [3], Gabriella Racchetti [3], Alessandro Podestà [1], Cristina Lenardi [1] and Paolo Milani [1,*]

[1] CIMaINa and Dipartimento di Fisica, Università degli Studi di Milano, Via Celoria 16, 20133 Milano, Italy; anita.previdi@unimi.it (A.P.); claudio.piazzoni@unimi.it (C.P.); francesca.borghi@unimi.it (F.B.); carsten.schulte@unimi.it (C.S.); alessandro.podesta@unimi.it (A.P.); cristina.lenardi@unimi.it (C.L.)

[2] Center for Materials and Microsystems (CMM), Bruno Kessler Foundation (FBK), via Sommarive 18, 38123 Trento, Italy; lorenzel@fbk.eu (L.L.); giaco@fbk.eu (F.G.); bucciarelli@fbk.eu (A.B.)

[3] Center for Behavioral Neuroscience and Communication (BNC), Università Vita-Salute San Raffaele, Via Olgettina 58, 20132 Milano, Italy; malgaroli.antonio@unisr.it (A.M.); lamanna.jacopo@hsr.it (J.L.); andrea.moro@unimi.it (A.M.); racchetti.gabriella@hsr.it (G.R.)

* Correspondence: paolo.milani@mi.infn.it

Citation: Previdi, A.; Piazzoni, C.; Borghi, F.; Schulte, C.; Lorenzelli, L.; Giacomozzi, F.; Bucciarelli, A.; Malgaroli, A.; Lamanna, J.; Moro, A.; et al. Micropatterning of Substrates for the Culture of Cell Networks by Stencil-Assisted Additive Nanofabrication. *Micromachines* **2021**, *12*, 94. https://doi.org/10.3390/12010094

Received: 2 January 2021
Accepted: 13 January 2021
Published: 18 January 2021

Publisher's Note: MDPI stays neutral with regard to jurisdictional claims in published maps and institutional affiliations.

Copyright: © 2021 by the authors. Licensee MDPI, Basel, Switzerland. This article is an open access article distributed under the terms and conditions of the Creative Commons Attribution (CC BY) license (http://creativecommons.org/licenses/by/4.0/).

AFM Analysis for the Assessment of Substrates and Anti-Fouling Treatment Protocol

Our approach requires glass substrates with low roughness, in order not to affect the nanostructured zirconia film roughness. We carried out an AFM roughness analysis on different commercial glass substrates, whose final surface polishing may differ, in order to select substrates with a roughness value below 1 nm.

Figures S1a,b show AFM images of two different commercial glass coverslips (Imglas and Zeus) with very different surface morphologies. Imglas coverslips (a) do not show significant modulations in terms of surface roughness on the scale of 2 nm and they have an overall roughness corresponding to 0.2 nm, whereas Zeus coverslips (b) have a rough appearance on the 10 nm z-scale and measurements report an overall roughness value of 4 nm. Imglas coverslips (a) were therefore selected.

The glass substrates are preliminary cleaned in an ultrasound bath (ultrasonic cleaning tank, purchased from Beta Professional tools, Sovico (MB), Italy) with an Alconox® detergent solution and then rinsed with ultrapure water (milli-Q). A further cleaning step with Piranha solution (H_2SO_4:H_2O_2 (3:1)) ensures the removal of any organic contaminant and allows the complete hydroxylation of the glass surface. Subsequently, the coverslips are thoroughly rinsed with ultrapure water and dried under pure N_2. All chemicals were purchased from Merck KGaA, Darmstadt, Germany.

After the cleaning procedure, three different anti-fouling protocols were tested for surface passivation:

(i) silanization by evaporation of dimethyldichlorosilane and subsequent deposition of Pluronic (3.5% for 1h) by drop-casting;
(ii) functionalization with the copolymer PAcrAm-g-(PMOXA, NH_2, Si) (produced by SuSoS AG 151 - Dubendorf, Switzerland [30,40]), by evaporation in static vacuum;
(iii) functionalization with the copolymer PAcrAm-g-(PMOXA, NH_2, Si), by immersion in a diluted solution (0.1 mg/mL in HEPES, pH 7.4) for 30 minutes.

The antifouling coating can influence the zirconia deposition, in particular if its roughness is not negligible with respect to the deposited film roughness.

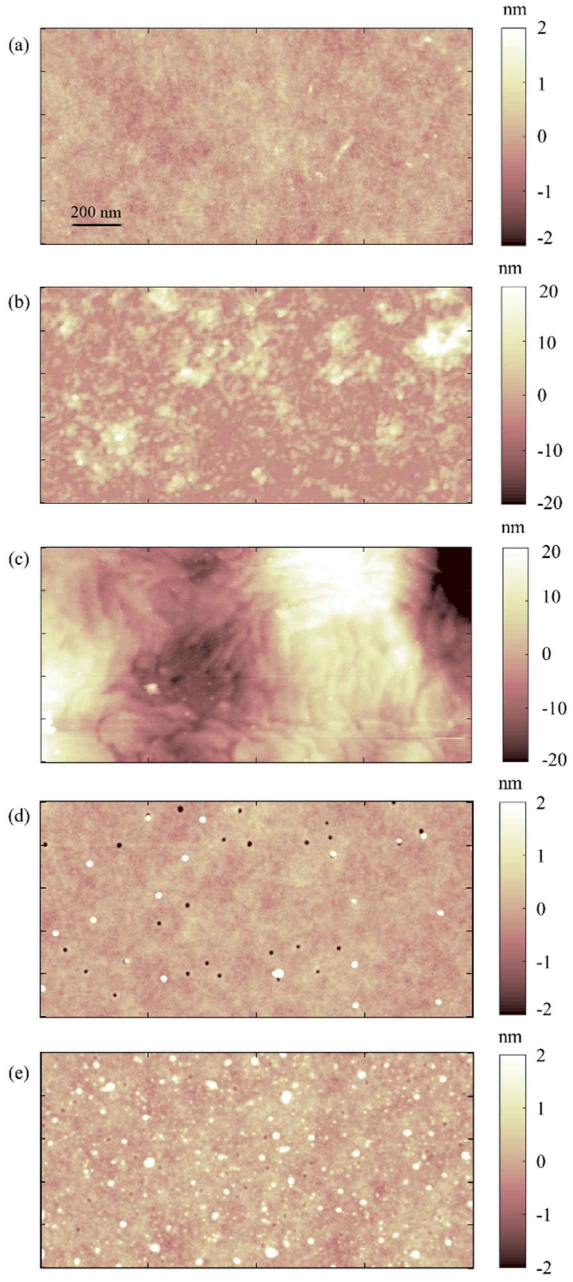

Figure 1. Morphological AFM maps (2μm × 1μm) of glass coverslips. (**a**) Pristine Imglas coverslip; (**b**) pristine Zeus coverslip; (**c**) Imglas coverslip passivated with Pluronic (method i); (**d**) Imglas coverslip passivated with evaporated copolymer PAcrAm-g-(PMOXA, NH_2, Si) (method ii); (**e**) Imglas coverslip passivated by immersion in solution of the copolymer PAcrAm-g-(PMOXA, NH_2, Si) (method iii).

In Figure S1c–e we show the AFM morphologies of coverslips treated with the three antifouling protocols described above. The pictures evidence a striking morphological difference between the glass surface treated with Pluronic (method i, Figure S1c) and those treated with PAcrAm-g-(PMOXA, NH_2, Si) (method ii and iii, Figure S1d,e). The measured R_q values reflect this difference: method (i) provides a surface with R_q ~10 nm, whereas for method (ii) and (iii) R_q ~ 0.5 nm. The copolymer PAcrAm-g-(PMOXA, NH_2, Si), assembles spontaneously in a monolayer that does not increase the surface roughness significantly. The final value of R_q measured below 1 nm can be neglected as it does not impact on the overall roughness of a nanostructured film.

The effectiveness of the antifouling properties of functionalized coverslips was confirmed by tests with PC12 cells and primary hippocampal neurons [29]. The best adhesion contrast between the patterned zirconia areas and the functionalized glass was obtained with method (iii). A time stability test of the antifouling layer performed with PC12 cells showed that the cell-repelling functionalization is effective for 4 weeks, at least.

Summary of Features of the Stencil Masks

We report a table summarizing the main features of all the different types of stencil masks tested in this work.

Table S1. Summary of the different types of masks tested in this work.

Code	Fabrication Technique	Material	Mask dimension (mm)	Thickness (µm)	Pattern type	Diameter of the dots (µm)	Center-center spacing (µm)
A	LC	Steel	14 × 15	150	dots	150	500
B	LC	Steel	14 ×15	150		150	300
C	LC	Steel	14 ×15	150		75	200
D	LC	Steel	14 ×15	150		50	100
Q10	LC	Steel	10.9 × 14	50	dots & channels (20 µm)	100	500
Q11	LC	Steel	10.9 × 14	50		100	1000
Q12	LC	Steel	10.9 × 14	50		100	1500
Q13	LC	Steel	10.9 × 14	50		250	500
Q14	LC	Steel	10.9 × 14	50		250	1000
Q15	LC	Steel	10.9 × 14	50		250	1500
Q16	PRL	Silicon	10.9 × 14	100	dots & channels (20/50 µm)	100	500
Q17	PRL	Silicon	10.9 × 14	100		100	1000
Q18	PRL	Silicon	10.9 × 14	100		100	1500
Q19	PRL	Silicon	10.9 × 14	100		250	500
Q20	PRL	Silicon	10.9 × 14	100		250	1000
Q21	PRL	Silicon	10.9 × 14	100		250	1500

Pattern Distortions

LC Steel Masks Defects

Fabrication defects in steel stencil masks can hamper the reproduction of micrometric patterns with SCBD.

Steel masks allow the experimenter to set up well defined and separate zirconia areas. In Figure S2a, a phase contrast image of nanostructured zirconia dots 150 µm wide obtained with a steel mask is reported. Figure S2 (b) shows the AFM height profile of one of the zirconia dots. The diameter of the homogeneous height area is only ~100 µm. The lateral rising widths are ~20 µm, consistent with the value expected of (17.5 ± 5.9) µm, calculated with equation (1), in the main text.

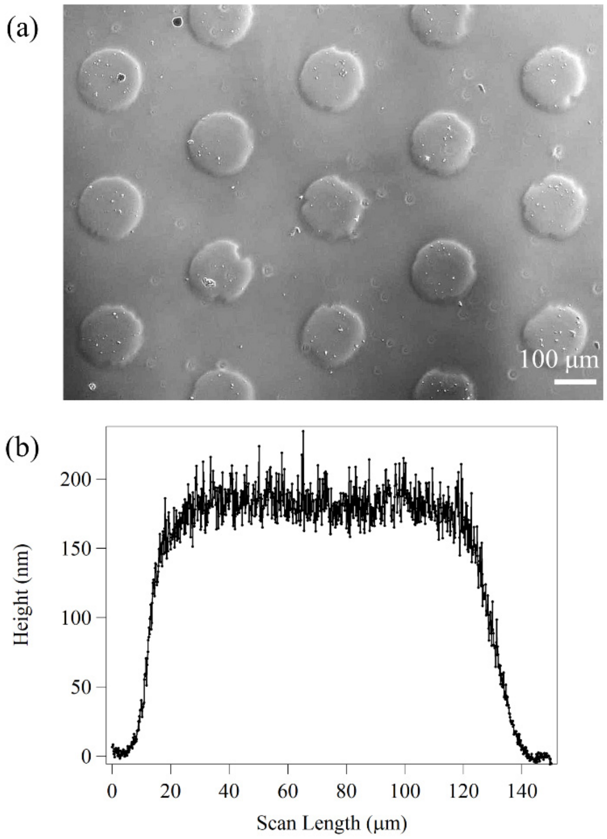

Figure S2. Nanostructured zirconia dots. (**a**) Phase contrast image of a patterned nanostructured zirconia film with 150 µm wide dots on a 300 µm hexagonal grid (mask A, Table S1). (**b**) AFM height profile map of a nanostructured zirconia dot.

With LC steel masks openings of few tens of micrometers, the effect of edge defects is disruptive. This is particularly evident, for example, in the pattern given by dots connected by 20 µm wide bridges reported in the phase contrast image of Figure S3.

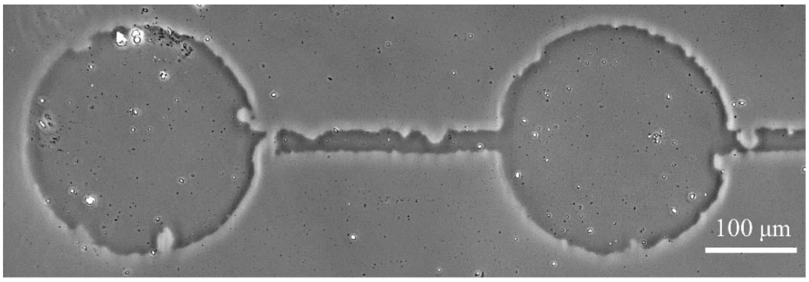

Figure S3. Ns-ZrO_x pattern reproduced with a steel LC mask with 250 µm wide dots and 20 µm wide channels (Q13, Table Scheme 1. The fabrication defects of the mask are transferred and amplified in the patterned film: the borders of the pattern feature are jagged. The micrometric bridge is not continuous since the dimension of the wiggles is comparable to its width.

Clogging—Cleaning of the Masks

The particles sticking on the edges of the openings of the masks can contribute to the distortion of the pattern, via clogging effect. This effect is particularly evident if the stencil mask pattern has features below few tens of micrometers and when the thickness of the deposit is not negligible with respect to the lateral dimensions of the openings. The latter case can be disregarded in the fabrication of zirconia films for biological application, as the typical thickness of the films deposited is below some hundreds of nanometers.

Instead, mask clogging may derive from an ineffective cleaning procedure after a deposition, if cluster aggregates get stuck into the mask openings. This effect may close them completely, compromising the pattern design.

Figure S4a,b reports scanning electron microscope (SEM) images of a poorly cleaned steel mask (ref Table S1, Q11), where the zirconia deposit blocks the mask openings. Figure S4c shows a deposited zirconia pattern obtained with poorly cleaned masks. This affects the pattern transfer: the microchannel is not continuous.

(a) (b) (c)

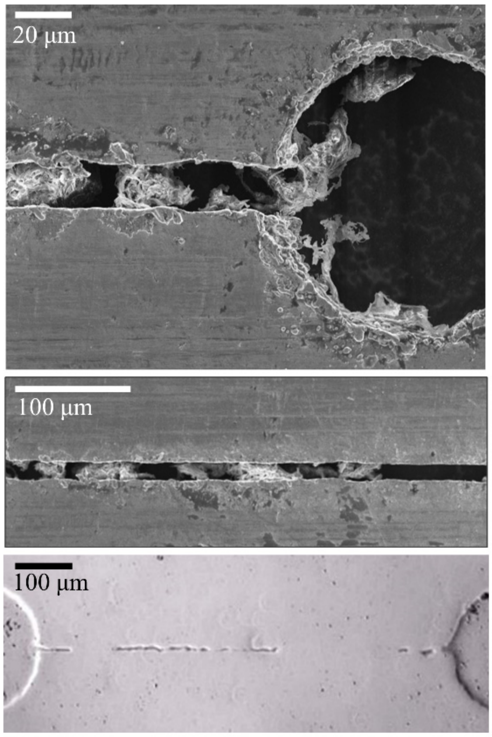

Figure 4. The clogging effect t (**a**,**b**) SEM images of LC stainless-steel masks (Q11, Table S1) not properly cleaned after a SCBD: the zirconia film portions completely block the masks opening, compromising the pattern. The images were acquired using a SEM (Zeiss Supra 40) with a 7 kV electron beam at different magnifications (10×–300×) and with a resolution of 0.5–1 nm/pixels. (**c**) Phase contrast image of a patterned zirconia film obtained with the stencil masks shown above. The result is a poor reproduction of the micrometric channel.

We found that masks can be efficiently cleaned by delicately removing the zirconia layer with wiping paper (Wypall 7300, Kimberly-Clark Professional). Possible leftover zirconia residuals can be completely removed with a 15-minute ultrasonic bath in EtOH. With this cleaning procedure the masks can be reused repeatedly for at least 20 cycles.
